# The clinical efficacy and safety of kanglaite adjuvant therapy in the treatment of advanced hepatocellular carcinoma: A PRISMA-compliant meta-analysis

**DOI:** 10.1042/BSR20193319

**Published:** 2019-11-26

**Authors:** Jingjing Liu, Xueni Liu, Jing Ma, Ke Li, Chao Xu

**Affiliations:** 1 Department of Infectious Disease, Liaocheng People’s Hospital, Liaocheng 252000, Shandong Province, China; 2 Department of Infectious Disease, Shandong Provincial Western Hospital, Jinan 250022, Shandong Province, China; 3 Department of Science and Education, Liaocheng People’s Hospital, Liaocheng 252000, Shandong Province, China; 4 Department of Central Laboratory, Liaocheng People’s Hospital, Liaocheng 252000, Shandong Province, China; 5 Department of Hepatobiliary Surgery, Liaocheng People’s Hospital, Liaocheng 252000, Shandong Province, China

**Keywords:** conventional treatments, hepatocellular carcinoma, kanglaite, meta-analysis, traditional Chinese medicine

## Abstract

Kanglaite, a type of Chinese medicine preparation, is considered a promising complementary therapy option for advanced hepatocellular carcinoma (HCC). Although an analysis of the published literature has been performed, the exact effects and safety are yet to be systematically investigated. Therefore, we conducted a wide-ranging online search of electronic databases to provide systematic conclusions; data from 31 trials with 2315 HCC patients were included. The results indicated that compared with conventional treatment (CT) alone, the combination of kanglaite with CT markedly prolonged patients’ 6-month overall survival (OS, *P*=0.003), 12-month OS (*P*<0.0001), 18-month OS (*P*=0.003), 24-month OS (*P*=0.03) and 36-month OS (*P*=0.0006) and significantly improved the overall response rate (odds ratio (OR) = 2.57, 95% confidence interval (CI) = 2.10–3.16, *P*<0.00001) and disease control rate (OR = 3.10, 95% CI = 2.42–3.97, *P*<0.00001) of patients. The quality of life (QoL), clinical symptoms and immune function of patients were also obviously improved after combined treatment. The incidence rates of nausea and vomiting (*P*=0.04), hepatotoxicity (*P*=0.0002), leukopenia (*P*<0.00001), thrombocytopenia (*P*<0.0001), gastrointestinal side effects (*P*=0.01) and fever (*P*<0.0009) were lower in the group receiving CT and kanglaite than in the group receiving CT alone. In summary, the combination of kanglaite and CT is safe and more effective in treating HCC than is CT alone, and its application in the clinic is worth promoting.

## Introduction

Hepatocellular carcinoma (HCC) is the third leading cause of cancer-related deaths, and in 2018, 781631 deaths worldwide were attributed to HCC [1]. Recently, the incidence of HCC has significantly increased, with approximately 840000 new cases every year [1]. China is a high-risk region for HCC, with the deaths caused by HCC in this country accounting for approximately 50% of HCC-related deaths worldwide [2]. HCC is a fatal disease with a poor prognosis. Despite the development of diagnostic methods, early detection of HCC remains difficult [3,4]. In most patients, HCC progresses to an advanced stage, with a 5-year survival rate of less than 20% [3]. Surgery and liver transplantation are regarded as the optimal treatment options, but only a small proportion of HCC patients can undergo potentially curative resection [3,4]. In addition, the therapeutic effects of current conventional treatment (CT), such as radiotherapy and chemotherapy for advanced HCC, are still unsatisfactory [3–5]. Therefore, effective comprehensive therapeutic approaches should be developed.

Traditional Chinese medicine has been widely applied as an effective complementary medicine for cancer treatment [5,6]. Kanglaite is an extract from Coix seeds, the main active ingredient of which is a triglyceride containing four types of fatty acids [7,8]. Kanglaite was formally approved in 1997 by the Ministry of Health of China for the treatment of malignancies such as HCC, non-small cell lung cancer (NSCLC) and pancreatic cancer (PC) [7,9,10]. Millions of cancer patients in numerous hospitals in China have been treated with kanglaite [7]. Moreover, kanglaite has shown good clinical efficacy in the U.S.A. It is also the first traditional Chinese medicine preparation approved by the U.S. Food and Drug Administration (FDA) for inclusion in clinical trials [11]. Yang et al. [8] demonstrated that kanglaite can effectively reverse the multidrug resistance (MDR) of human HCC and enhance the sensitivity of tumor cells to chemotherapeutic drugs by inducing apoptosis and cell cycle arrest via the PI3K/AKT pathway. Moreover, Huang et al. [12]. found that kanglaite can inhibit HepG2 cell transplantation-induced tumor growth by stimulating anticancer immune responses. In addition, kanglaite can induce cancer cell apoptosis by activating proapoptotic factors, such as p53, Fas and caspase-3 [13,14].

**Figure 1:**
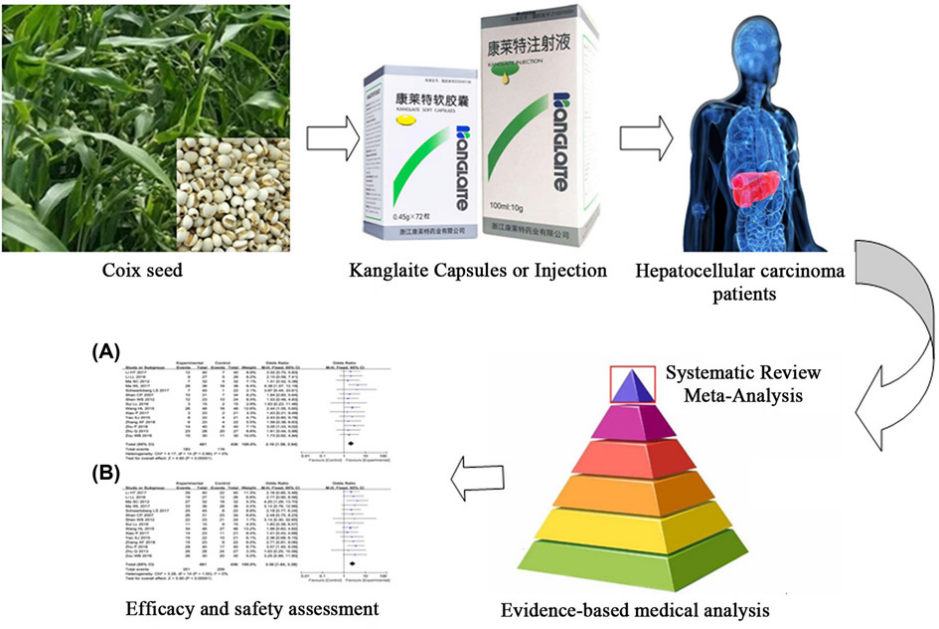
Work flow of the present study (**A**) Efficacy (**B**) Safety.

Several studies have indicated that CT combined with kanglaite exhibits more prominent therapeutic effects for advanced HCC than does CT alone [10]. In a meta-analysis comparing hepatic arterial intervention combined with kanglaite and hepatic arterial intervention alone, the former had a significantly higher overall response rate (ORR), though the outcomes discussed were not complete. In fact, overall survival (OS), the disease control rate (DCR), quality of life (QoL), clinical symptoms, immune function and safety were not considered in that analysis [10]. Moreover, the small sample size included may have influenced the analysis of therapeutic effects. Therefore, in the present study, we conducted an up-to-date meta-analysis to investigate the clinical efficacy and safety of CT combined with kanglaite in comparison with CT alone for the treatment of advanced HCC (Figure 1) to provide a scientific basis for the design of future clinical trials.

## Materials and methods

This systematic review and meta-analysis was performed following the Preferred Reporting Items for Systematic Reviews (PRISMA) guidelines and Cochrane Handbook. Ethical approval was not necessary because the present study was a meta-analysis.

### Search strategy and selection criteria

Nine electronic databases, namely, PubMed, Cochrane Library, Web of Science, Embase, Medline, China National Knowledge Infrastructure (CNKI), Wanfang Database, Chinese Scientific Journal Database (VIP) and Chinese Biological Medicine Database (CBM), were searched up to May 2019 using the key terms ‘kanglaite’ or ‘kanglaite injection’ or ‘kanglaite capsule’ or ‘coix seed capsule’ or ‘coix seed injection’ combined with ‘hepatocellular carcinoma’ or ‘hepatocellular cancer’ or ‘hepatocellular tumor’ or ‘liver carcinoma’ or ‘liver cancer’ or ‘hepatocellular tumor’ (Supplementary Table S1).

The inclusion criteria were as follows: (1) controlled trials with advanced HCC patients; (2) studies involving more than 30 HCC patients; (3) studies comparing the clinical outcomes of CT plus kanglaite adjuvant therapy (experimental group) with those of CT alone (control group); and (4) the CT included transcatheter arterial chemoembolization (TACE), transhepatic arterial embolization (TAE), chemotherapy, stereotactic radiotherapy (SRT), support and symptomatic treatment (SST) and targeted therapy.

The exclusion criteria were as follows: (1) patients with mixed malignancies; (2) articles without sufficient available data; and (3) noncontrast articles, case studies and review papers.

### Data extraction and quality assessment

Data were independently extracted by two reviewers (Jingjing Liu and Xueni Liu) according to the above inclusion and exclusion criteria; disagreements were adjudicated by the third investigator (Chao Xu). The data extracted comprised the following items: (a) the first author’s name; (b) year of publication; (c) tumor stages or Karnofsky performance score (KPS); (d) number of cases; (e) therapeutic regimens; (f) dosage of kanglaite; and (g) study parameters. To ensure the quality of the meta-analysis, the quality of the included randomized and nonrandomized controlled trials was evaluated according to the Cochrane Handbook tool [15] and Methodological Index for Nonrandomized Studies (MINORS, Supplementary Table S2), respectively [16].

### Outcome definition

The clinical responses assessed included treatment efficacy, QoL, clinical symptoms, immune function and adverse events. Treatment efficacy was evaluated in terms of the OS rate, ORR and DCR. QoL was assessed using the KPS scale. The clinical symptoms of the patients included the following indicators: appetite, hepatalgia, abdominal distension, fatigue and jaundice. Immune function indicators (percentages of CD3^+^, CD4^+^, CD8^+^ and NK cells and the CD4^+^/CD8^+^ ratio) and the decrease rate of α-fetoprotein (AFP) in HCC patients were determined and compared between the kanglaite and nonkanglaite groups. Adverse events, including nausea and vomiting, hepatotoxicity, nephrotoxicity, leukopenia, thrombocytopenia gastrointestinal adverse effects, anemia, fever, myelosuppression and alopecia, were also assessed.

### Statistical analysis

Statistical analysis was performed with RevMan 5.3 (Nordic Cochran Centre, Copenhagen, Denmark) and Stata 13.0 (Stata Corp., College Station, TX, U.S.A.) software. All data are expressed as odds ratios (ORs) and 95% confidence intervals (CIs), and *P*<0.05 indicated a significant difference. Heterogeneity among the studies was assessed by Cochran’s Q test; *I^2^* < 50% or *P*>0.1 indicated a lack of heterogeneity among the studies [17]. When the level of heterogeneity was small (*I^2^* < 50%), a fixed-effects model was applied for OR estimation; otherwise, a random-effects model was selected.

Publication bias was analyzed with Begg’s and Egger’s regression tests, and the results are presented in funnel plots. Pooled analysis of publication bias determined that the trim-and-fill method should be applied to coordinate the estimates from unpublished studies; the adjusted results were compared with the original pooled OR [18,19]. Sensitivity analysis was conducted to evaluate the impacts of different therapeutic regimens, kanglaite dosages, sample sizes and research types on clinical efficacy.

## Results

### Search results

**Figure 2:**
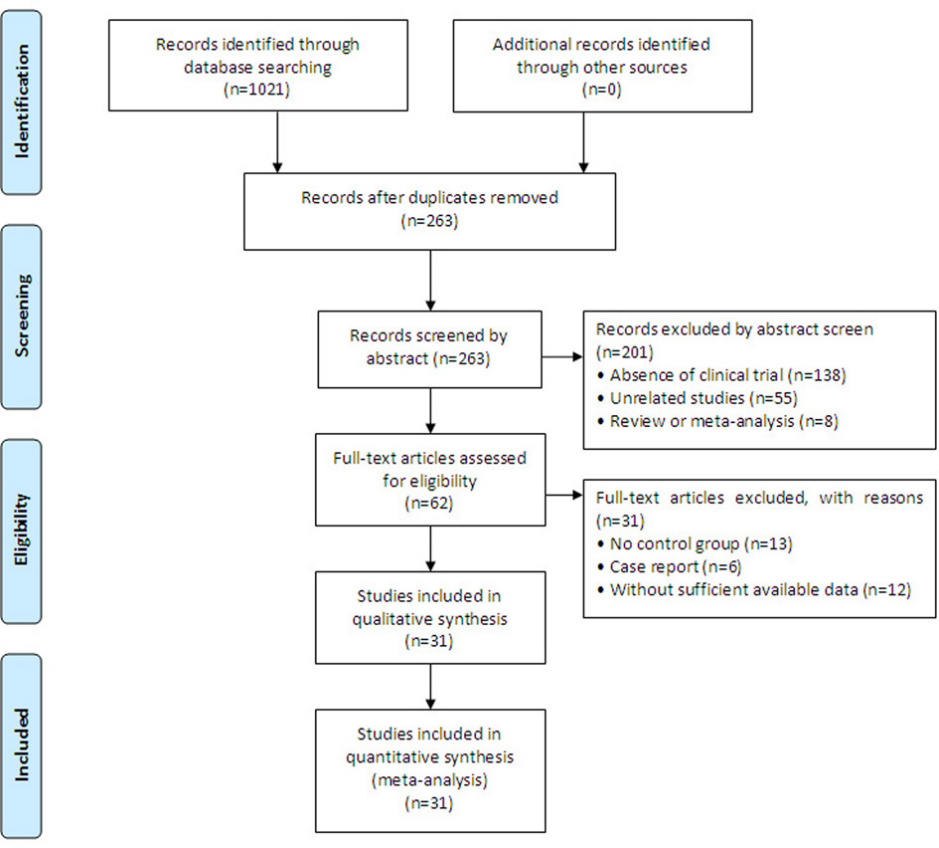
Study selection process for the meta-analysis

In total, 1021 articles were initially identified. Of those, 758 papers were excluded because they were duplicates. After title and abstract review, 201 articles were further excluded because they were not clinical trials (*n*=138), were unrelated studies (*n*=55), or were reviews or meta-analyses (*n*=8), leaving 62 studies that were potentially relevant. After a detailed assessment of the full text articles, those without a control group (*n*=13), studies that were case reports (*n*=6), and trials with insufficient data (*n*=12) were excluded. Ultimately, 31 trials [20–50] involving 2315 advanced HCC patients were included in this analysis (Figure 2).

### Patient characteristics

All included trials were performed in different medical centers in China. In total, 1219 advanced HCC patients were treated with CT combined with kanglaite adjuvant therapy; 1096 patients were treated with CT alone. All included trials except one [27] clearly stated the dosage of kanglaite administered. Detailed information on the involved studies and HCC patients is shown in Table 1. The kanglaite used was manufactured by Zhejiang Kanglaite Pharmaceutical Co., Ltd. The Quality Standards of kanglaite in the present study were approved by the Chinese State Food and Drug Administration (SFDA) and were granted a Manufacturing Approve Number issued by Chinese SFDA (Z20040138 and Z10970091). All pharmaceutical companies involved followed the quality processing procedure outlined in Chinese Pharmacopeia.

**Table 1:** Clinical information from the eligible trials in the meta-analysis

Included studies	Tumor stage/KPS	Patients Con/Exp	Therapeutic regimen		Dosage of kanglaite	Parameter types
			Experimental	Control (drugs)		
Ao, M. (2017)	≥60	38/38	Con+ kanglaite^1^	TACE (DDP, 5-Fu, E-ADM)	20 g/time, 1 time/day	ORR, DCR, AE
Feng, Y.Z. (2001)	II–IV	21/11	Con+KLT^1^	TAE	20 g/time, 1 time/day	ORR, DCR, IF
Hu, J.B. (2003)	II–IV	25/31	Con+KLT^1^	TACE (DDP, 5-Fu, THP)	20 g/time, 1 time/day	ORR, DCR, AE, CS, AFP, QoL
Jiang, Y.B. (2006)	I–III	51/105	Con+KLT^1^	TACE (DDP, 5-Fu, ADM)	20 g/time, 1 time/day	ORR, DCR, CS, QoL
Li, D.J. (2009)	II–III	32/30	Con+KLT^2^	TACE (DDP, 5-Fu, ADM)	2.7 g /time, 4 times/day	OS, ORR, DCR, AE, AFP, QoL
Li, M. (2015)	II-III	23/24	Con+KLT^1^	CT (Oxaliplatin)	20 g/time, 1 time/day	OS, ORR, DCR, IF, CS, QoL
Li, Y. (2014)	I–IV	75/75	Con+KLT^1^	CT (Meccnu, ADM, 5-Fu)	20 g/time, 1 time/day	OS, ORR, DCR, AE, QoL
Liang, S.M. (2006)	II-III	25/31	Con+KLT^1^	TAE	unknown	ORR, DCR, CS, AFP
Lu, D.P. (2017)	Unknown	43/51	Con+KLT^1^	TACE (Oxaliplatin, 5-Fu)	20 g/time, 1 time/day	OS, ORR, DCR, QoL
Lu, H. (2006)	I–III	24/24	Con+KLT^1^	TACE (DDP, 5-Fu, MMC)	20 g/time, 1 time/day	OS, ORR, DCR, AE, QoL
Lv, D.Z. (2004)	II–III	38/38	Con+KLT^1^	TACE (unknown), SST	20 g/time, 1 time/day	IF, CS, QoL
Ma, W.L. (2017)	Unknown	43/43	Con+KLT^1^	CT (FOLFOX)	20 g/time, 1 time/day	ORR, DCR, IF
Qin, G.Y. (1998)	Unknown	20/18	Con+KLT^1^	TACE (DDP, 5-Fu, THP)	10-20 g/time, 1 time/day	ORR, DCR, CS
Qin, Y.T. (2001)	Unknown	42/52	Con+KLT^1^	SST	20 g/time, 1 time/day	IF
Shao, L. (2017)	II–III	25/25	Con+KLT^1^	SRT	20 g/time, 1 time/day	ORR, AE, QoL
Wang, C.H. (2001)	I–III	50/50	Con+KLT^1^	TACE (DDP, ADM, HCPT)	10 g/time, 1 time/day	ORR, DCR
Wang, X.F. (2012)	III–IV	24/34	Con+KLT^1^	TACE (unknown) FOLFOX	10 g/time, 1 time/day	ORR, AE, QoL
Wei, Q.C. (2009)	Unknown	24/24	Con+KLT^1^	SST	10 g/time, 1 time/day	QoL
Wu, D.H. (2009)	II–III	30/30	Con+KLT^1^	CT (Oxaliplatin, FUDR)	20 g/time, 1 time/day	ORR, DCR, AE, CS, QoL
Wu, J.L. (2015)	Unknown	60/60	Con+KLT^1^	TACE (unknown)	10 g/time, 1 time/day	ORR, DCR, QoL
Xi, D.S. (2001)	I–III	20/20	Con+KLT^1^	CT (E-ADM, 5-Fu, HCPT, ACTD)	20 g/time, 1 time/day	ORR, DCR, AE
Xu, J. (2018)	Unknown	54/54	Con+KLT^1^	CT (Meccnu, ADM, 5-Fu)	20 g/time, 1 time/day	ORR, DCR
Xu, X.H. (2010)	II–III	37/38	Con+KLT^1^	CT (Capecitabine)	20 g/time, 1 time/day	OS, ORR, DCR, AE, CS
Yang, T. (2013)	≥60	30/60	Con+KLT^1^	TACE (DDP, 5-Fu, E-ADM)	10 g/time, 1 time/day	ORR, DCR, AE, CS, QoL
Ye, X. (2003)	III-IV	17/19	Con+KLT^1^	TACE (DDP, 5-Fu, ADM, MMC)	20 g/time, 1 time/day	ORR, DCR, AE, CS, AFP, QoL
Yin, R.R. (2009)	Unknown	32/40	Con+KLT^1^	TACE (unknown)	10 g/time, 1 time/day	ORR, DCR
Yu, Z.H. (2016)	≥50	20/20	Con+KLT^1^	Thalidomide	20 g/time, 1 time/day	OS, ORR, DCR, AE, QoL
Zhang, Y. (2012)	>50	31/31	Con+KLT^1^	SST	10 g/time, 1 time/day	AFP, QoL
Zhang, Y.J. (2017)	II–III	48/49	Con+KLT^1^	TACE (DDP, 5-Fu, ADM, MMC)	20 g/time, 1 time/day	ORR, DCR, AE, AFP, QoL
Zhou, S.F. (2018)	III–IV	54/54	Con+KLT^1^	Sorafenib	20 g/time, 1 time/day	ORR, DCR, IF
Zhu, X.F. (2006)	I–IV	40/40	Con+KLT^1^	TACE (DDP, 5-Fu, THP)	20 g/time, 1 time/day	ORR, DCR, CS, QoL

Con, control group (CTs alone group); Exp, experimental group (CTs and kanglaite group). Abbreviations: ACTD, actinomycin D; ADM, adriamycin; AE, adverse event; CF, calcium folinate; CS, clinical symptom; DDP, cisplatin; E-ADM, epirubicin; FOLFOX, qxaliplatin+CF+5-Fu; HCPT, hydroxycamptothecin; IF, immune function; MMC, mitomycin C; ORR, overall response rate; THP, pirarubicin; 5-Fu, 5-Fluorouracil. ^1^Kanglaite injection. ^2^Kanglaite capsules.

### Quality assessment

**Figure 3:**
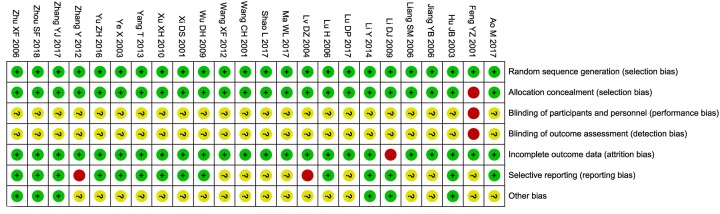
Risk of bias summary: review of the authors’ judgments about each risk of bias item for the included randomized controlled studies Each color represents a different level of bias: red indicates high risk, green indicates low risk and yellow indicates an unclear risk of bias.

The quality assessment of the risk of bias is shown in Figure 3 and Supplementary Table S3. The results showed that the literature recruited in the present study was of good quality.

### Therapeutic efficacy assessments

**Figure 4:**
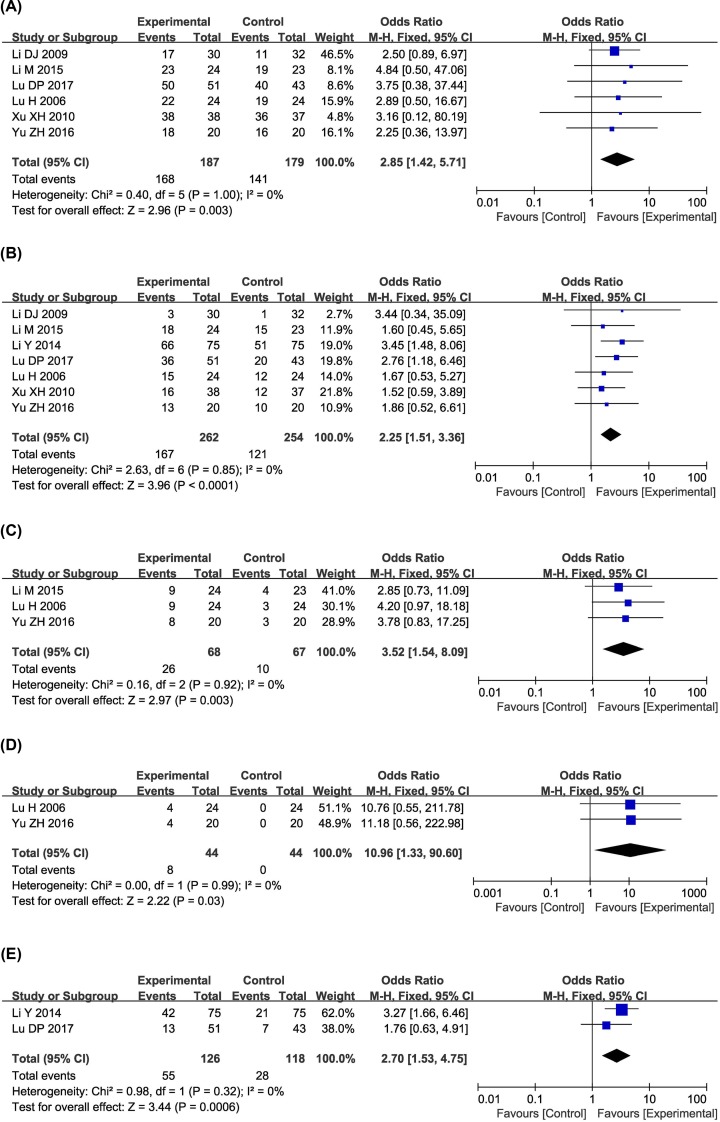
Comparisons of OS between control and experimental group Forest plot of the comparison of 6-month (**A**); 12-month (**B**); 18-month (**C**); 24-month (**D**); and 36-month (**E**), OS between the experimental and control groups. Control group, CT alone group; experimental group, CTs and kanglaite group. A fixed effects meta-analysis model (Mantel–Haenszel method) was used.

**Figure 5:**
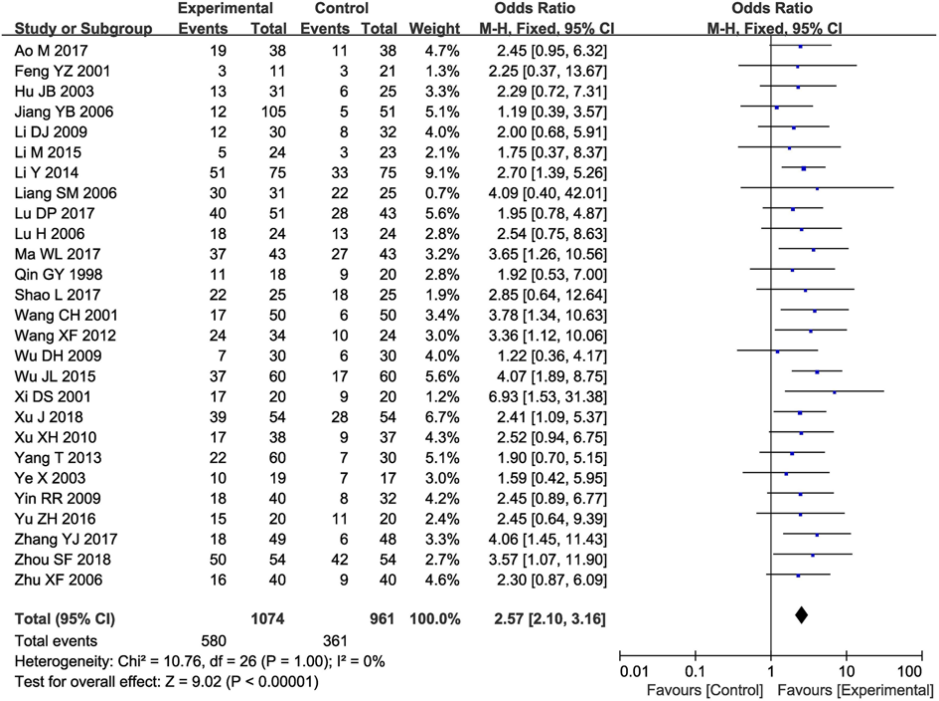
Forest plot of the comparison of overall response rates between the experimental and control groups Control group, CTs alone group; experimental group, CTs and kanglaite group. A fixed effects meta-analysis model (Mantel–Haenszel method) was used.

**Figure 6:**
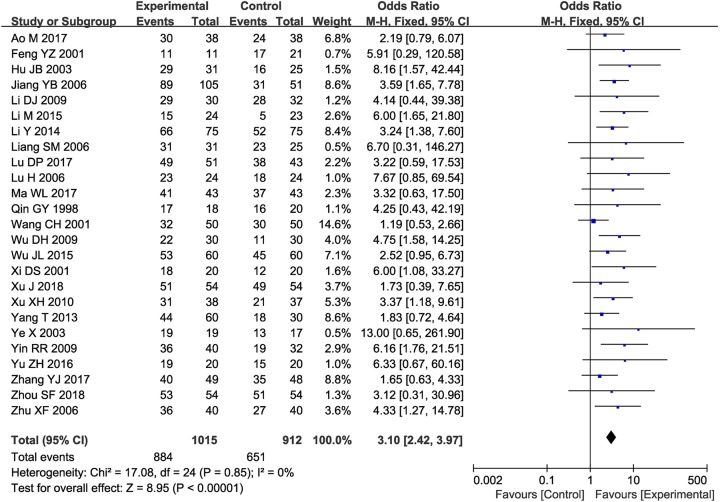
Forest plot of the comparison of DCRs between the experimental and control groups Control group, CTs alone group; experimental group, CTs and kanglaite group. A fixed effects meta-analysis model (Mantel–Haenszel method) was used.

As shown in Figures 4–6, pooled results showed that compared with those who underwent CT alone, patients who underwent combined therapy had significantly improved 6-, 12-, 18-, 24- and 36-month OS (6-month OS: OR = 2.85, 95% CI = 1.42–5.71, *P*=0.003; 12-month OS: OR = 2.25, 95% CI = 1.51–3.36, *P*<0.0001; 18-month OS: OR = 3.52, 95% CI = 1.54–8.09, *P*=0.003; 24-month OS: OR = 10.96, 95% CI = 1.33–90.60, *P*=0.03; 36-month OS: OR = 2.70, 95% CI = 1.53–4.75, *P*=0.0006), ORR (OR = 2.57, 95% CI = 2.10–3.16, *P*<0.00001) and DCR (OR = 3.10, 95% CI = 2.42–3.97, *P*<0.00001). Fixed-effect models were applied to analyze the OR rate because of the low degree of heterogeneity.

### Detection of AFP

**Figure 7:**
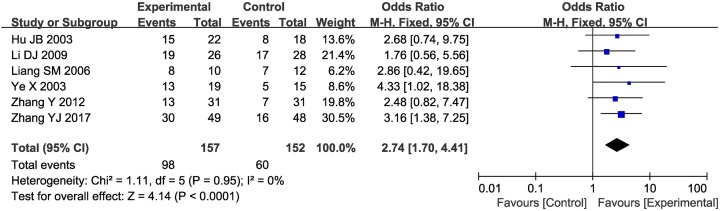
Forest plot of the comparison of the AFP decrease rate between the experimental and control groups Control group, CTs alone group; experimental group, CTs and kanglaite group. A fixed effects meta-analysis model (Mantel–Haenszel method) was used.

Six clinical trials [22,24,27,44,47,48] with 369 patients reported data on the AFP decrease rate between the two groups. As shown in Figure 7, the AFP decrease rate was significantly lower in patients receiving the combination treatment than in those receiving the CT alone (OR = 2.74, 95% CI = 1.70–4.41, *P*<0.0001). As no obvious heterogeneity was found among the included articles, a fixed-effects model was used to pool data.

### QoL assessment

**Figure 8:**
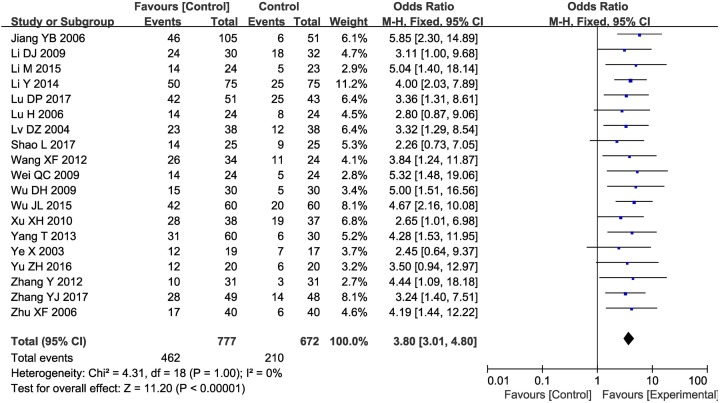
Forest plot of the comparison of QoL scores between the experimental and control groups Control group, CTs alone group; experimental group, CTs and kanglaite group. A fixed effects meta-analysis model (Mantel–Haenszel method) was used.

Nineteen trials [23–26,28–30,34,36–39,42–44,46–48,50] with 1449 patients reported QoL according to the KPS scale (Figure 8). According to the results, the QoL of HCC patients in the combined group was significantly better than that of patients in the control group (OR = 3.80, 95% CI = 3.01–4.80, *P*<0.00001). A fixed-effect model was used due to the low level of heterogeneity.

### Assessment of clinical symptoms

The clinical symptoms of HCC patients receiving combined therapy were significantly improved compared with those of patients treated with CT alone (Supplementary Figure S1, OR = 5.36, 95% CI = 3.21–8.94, *P*<0.00001), as indicated by increased appetite and reductions in hepatalgia, abdominal distension, fatigue and jaundice (Supplementary Figure S1, appetite: OR = 5.50, 95% CI = 1.72–17.61, *P*=0.004; hepatalgia: OR = 2.95, 95% CI = 1.74–5.00, *P*<0.0001; abdominal distension: OR = 3.52, 95% CI = 1.33–9.31, *P*=0.01; fatigue: OR = 4.60, 95% CI = 1.89–11.22, *P*=0.0008; jaundice: OR = 1.42, 95% CI = 0.41–4.95, *P*=0.59), though the improvement in jaundice was not significant.

### Immune function evaluation

**Figure 9:**
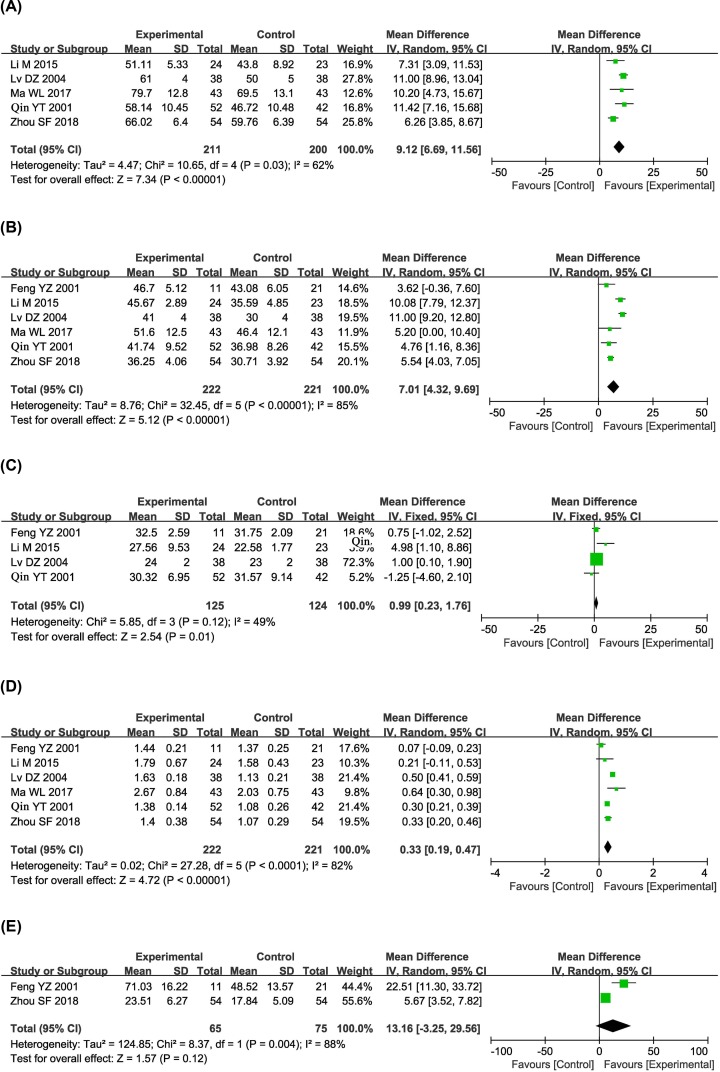
Comparisons of immune function between control and experimental group Forest plot of the comparison of immune function (CD3^+^ (**A**); CD4^+^ (**B**); CD8^+^ (**C**); CD3^−^CD56^+^ (**D**); and CD4^+^/CD8^+^ (**E**)) between the experimental and control groups. Control group, CTs alone group; experimental group, CTs and kanglaite group.

The immune status of patients between kanglaite and nonkanglaite groups was examined in six controlled studies [21,25,30,31,33,49]. As presented in Figure 9, the percentages of CD3^+^, CD4^+^ and CD8^+^ cells and the CD4^+^/CD8^+^ ratio were significantly higher in the combined treatment group than in the control group (CD3^+^: OR = 9.12, 95% CI = 6.69–11.56, *P*<0.00001; CD4^+^: OR = 7.01, 95% CI = 4.32–9.69, *P*<0.00001; CD8^+^: OR = 0.99, 95% CI = 0.23–1.76, *P*=0.01; CD4^+^/CD8^+^: OR = 0.33, 95% CI = 0.19–0.47, *P*<0.00001). However, the proportions of NK (CD3^−^CD56^+^) cells did not differ significantly between the two groups (OR = 13.16, 95% CI = −3.25–29.56, *P*=0.12). The percentage of CD8^+^ cells was not heterogeneous among the studies; thus, a fixed-effect model was used to analyze the OR. Otherwise, random-effects models were used.

### Assessment of adverse events

As shown in Table 2 and Supplementary Figure S2, compared with patients treated with CT alone, those treated with kanglaite and CT displayed lower incidence rates of nausea and vomiting [24,26,34,36,42,48,50], hepatotoxicity [20,22,24,34,36,44,50] leukopenia [22,34,44,48,50], thrombocytopenia [22,34,49,50], gastrointestinal side effects [20,25,29,43,44,46] and fever [24,44,48] (nausea and vomiting: OR = 0.62, 95% CI = 0.39–0.97, *P*=0.04; hepatotoxicity: OR = 0.40, 95% CI = 0.25–0.66, *P*=0.0002; leukopenia: OR = 0.28, 95% CI = 0.17–0.47, *P*<0.00001; thrombocytopenia: OR = 0.21, 95% CI = 0.10–0.42, *P*<0.0001; gastrointestinal side effects: OR = 0.43, 95% CI = 0.22–0.84, *P*=0.01; fever: OR = 0.37, 95% CI = 0.20–0.66, *P*=0.0009). In contrast, the incidence rates of nephrotoxicity [22,44,50], anemia [22,50], myelosuppression [26,43] and alopecia [26,49] (nephrotoxicity: OR = 0.16, 95% CI = 0.01–3.56, *P*=0.25; anemia: OR = 0.75, 95% CI = 0.33–1.73, *P*=0.50; myelosuppression: OR = 0.64, 95% CI = 0.34–1.19, *P*=0.16; alopecia: OR = 0.64, 95% CI = 0.34–1.24, *P*=0.19) did not differ significantly between the two groups. Fixed-effect models were used in these analyses due to the low level of heterogeneity.

**Table 2:** Comparison of adverse events between the experimental and control groups

Adverse events	Experimental group	Control group	Analysis method	Heterogeneity		OR	95% CI	*P*-value
	Number of patients (*n*) ref	Number of patients (*n*) ref		*I^2^* (%)	*P*-value			
Nausea and vomiting	291	281	Fixed	0	0.53	0.62	0.39–0.97	0.04
Hepatotoxicity	217	201	Fixed	0	0.70	0.40	0.25–0.66	0.0002
Nephrotoxicity	90	82	Fixed	─	─	0.16	0.01–3.56	0.25
Leukopenia	164	155	Fixed	0	0.94	0.28	0.17–0.47	<0.00001
Thrombocytopenia	150	144	Fixed	0	0.79	0.21	0.10–0.42	<0.0001
Gastrointestinal adverse effects	185	152	Fixed	0	0.64	0.43	0.22–0.84	0.01
Anemia	71	65	Fixed	0	0.64	0.75	0.33–1.73	0.50
Fever	98	97	Fixed	10	0.33	0.37	0.20–0.66	0.0009
Myelosuppression	135	105	Fixed	0	0.71	0.64	0.34–1.19	0.16
Alopecia	129	129	Fixed	0	0.72	0.64	0.34–1.24	0.19

Control group, CTs alone group; Experimental group, CTs and kanglaite group.

### Publication bias

**Figure 10:**
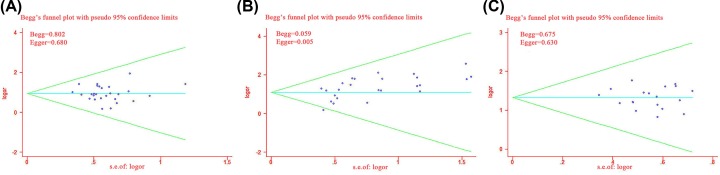
Funnel plot of publication bias Funnel plot of the ORR (**A**); DCR (**B**); and QoL (**C**).

Publication bias was assessed visually with funnel plots. As illustrated in Figure 10, the funnel plots were symmetrical for ORR and QoL but asymmetrical for DCR.

We also assessed publication bias by Begg’s and Egger’s regression tests, and DCR was found to have bias (Begg = 0.059; Egger = 0.005). Conversely, no significant publication bias was found for ORR (Begg = 0.802; Egger = 0.680) or QIR (Begg = 0.675; Egger = 0.630). To determine if the bias affected the pooled risk for DCR, we conducted a trim-and-fill analysis. The adjusted OR indicated the same trend as was indicated by the result of the primary analysis (before: *P*<0.0001, after: *P*<0.0001), reflecting the reliability of our primary conclusions.

### Sensitivity analysis

A sensitivity analysis was conducted, and one trial [24] was excluded because the type of kanglaite was in capsule form in the present study. The results of this analysis were similar to those obtained from the overall analysis of the pooled trials.

To explore the sources of ORR, DCR and QoL heterogeneity, we also conducted subgroup analyses with respect to therapeutic regimen, kanglaite dosage, sample size and type of study. As shown in Table 3, our analysis revealed no significant differences between different dosages of kanglaite, sample sizes and types of studies. Moreover, our results showed that kanglaite increased ORR and DCR among HCC patients only when combined with TACE/CT regimens.

**Table 3:** Subgroup analyses of ORR, DCR and QoL between the experimental and control groups

Parameter	Factors at study level	Experimental group	Control group	Analysis method	Heterogeneity		OR	95% CI	*P*-value
		Number of patients (*n*) ref	Number of patients (*n*) ref		*I^2^* (%)	*P*-value	(OR)		
ORR	**Therapeutic regimen**								
	kanglaite+TACE	649	534	Fixed	0	0.95	2.49	1.91–3.25	<0.00001
	kanglaite+CT	284	282	Fixed	0	0.71	2.62	1.81–3.78	<0.00001
	kanglaite+TAE	42	46	Fixed	0	0.69	2.88	0.71–11.78	0.14
	**Dosage of kanglaite**								
	200 ml/day	751	688	Fixed	0	0.98	2.47	1.93–3.15	<0.00001
	100 ml/day	244	196	Fixed	0	0.78	3.09	2.01–4.73	<0.00001
	**Study sample size**								
	>60	787	687	Fixed	0	0.96	2.64	2.07–3.36	<0.00001
	≤60	287	274	Fixed	0	0.96	2.41	1.63–3.56	<0.0001
	**Type of control trials**								
	RCT	878	772	Fixed	0	0.99	2.53	2.01–3.20	<0.00001
	Non-RCT	196	189	Fixed	0	0.78	2.72	1.76–4.21	<0.00001
DCR	**Therapeutic regimen**								
	kanglaite+TACE	615	510	Fixed	0	0.48	2.74	2.02–3.72	<0.00001
	kanglaite+CT	284	282	Fixed	0	0.90	3.71	2.36–5.82	<0.00001
	kanglaite+TAE	42	46	Fixed	0	0.95	6.26	0.72–54.47	0.10
	**Dosage of kanglaite**								
	200 ml/day	726	663	Fixed	0	0.96	3.56	2.63–4.81	<0.00001
	100 ml/day	210	172	Fixed	40	0.17	2.07	1.30–3.30	0.002
	**Study sample size**								
	>60	787	687	Fixed	0	0.80	2.58	1.95–3.40	<0.00001
	≤60	228	225	Fixed	0	1.00	6.14	3.47–10.88	<0.00001
	**Type of control trials**								
	RCT	819	723	Fixed	0	0.79	2.99	2.28–3.94	<0.00001
	Non-RCT	196	189	Fixed	0	0.60	3.59	2.01–6.40	<0.0001
QoL	**Therapeutic regimen**								
	kanglaite+TACE	510	407	Fixed	0	0.99	3.81	2.83–5.14	<0.00001
	kanglaite+CT	167	165	Fixed	0	0.82	3.88	2.43–6.20	<0.00001
	kanglaite+SST	55	55	Fixed	0	0.85	4.89	1.89–12.61	0.001
	**Dosage of kanglaite**								
	200 ml/day	538	471	Fixed	0	0.99	3.62	2.75–4.78	<0.00001
	100 ml/day	209	169	Fixed	0	1.00	4.48	2.80–7.17	<0.00001
	**Study sample size**								
	>60	577	485	Fixed	0	0.99	3.89	2.94–5.14	<0.00001
	≤60	200	187	Fixed	0	0.96	3.58	2.33–5.50	<0.00001
	**Type of control trials**								
	RCT	669	565	Fixed	0	1.00	3.63	2.81–4.68	<0.00001
	Non-RCT	108	107	Fixed	0	0.98	4.88	2.71–8.76	<0.00001

Control group, CTs alone group; Experimental group, CTs and kanglaite group. Abbreviation: kanglaite, Kanglaite.

## Discussion

The disadvantages of current CT for malignancies, such as drug resistance and toxic side effects, are a substantial burden for cancer patients [3,5]. Clinicians have been exploring complementary and alternative treatments to improve patients’ survival time, QoL and immune function and to reduce side effects caused by radiochemotherapy [3,5,10]. Kanglaite, a type of traditional Chinese medicine, has been clinically applied as an adjuvant therapy for decades [12,51]. Many studies have reported that the addition of kanglaite may be beneficial for HCC patients [14]. Although statistical analyses of the published literature have been performed, the exact therapeutic effects have not been systematically investigated. In this analysis, we conducted a wide-ranging online search with strict inclusion and exclusion criteria to provide clear and systematic conclusions.

The meta-analysis was performed with 27 articles [20–29,31,32,34–36,38–46,48–50] to evaluate the clinical efficacy of the addition of kanglaite to CT. Our analysis found that compared with CT alone, the combination of kanglaite and CT significantly improved survival time at 6, 12, 18, 24 and 36 months (*P*<0.05), suggesting that the addition of kanglaite to CT might prolong the survival time of HCC patients with advanced disease. The analysis considered ORR, DCR, QoL and clinical symptoms, all of which showed significant improvements in the combined group compared with the control group. Moreover, AFP is commonly used to predict the recurrence, metastasis and prognosis of HCC after comprehensive treatments [52,53], and our analysis showed that AFP was clearly reduced after treatment with the combination of CT and kanglaite. All these results indicate that using kanglaite might enhance the curative effects of CT for advanced HCC.

The immunosuppressed status of cancer patients has been reported, and immune system reconstruction is a critical approach for effectively treating malignancies. Our analysis showed that the percentages of CD3^+^, CD4^+^ and CD8^+^ cells and the CD4^+^/CD8^+^ ratio were significantly increased when kanglaite was administered to HCC patients, indicating that the immune function of HCC patients was improved by kanglaite-mediated therapy.

The meta-analysis evaluated the incidence rates of side effects after therapy, clearly showing reductions in nausea and vomiting, hepatotoxicity, leukopenia, thrombocytopenia, gastrointestinal side effects and fever (*P*<0.05) in the combined group compared with the control group. Therefore, kanglaite is a safe auxiliary antitumor medicine for advanced HCC and can effectively alleviate some of the adverse events associated with CT.

This analysis of therapeutic effects may have been influenced by several factors. In our study, no differences were found between different dosages of kanglaite, sample sizes and research types. Moreover, the results of subgroup analyses indicated that kanglaite increased HCC patient ORR and DCR only when combined with TACE/CT regimens. Nonetheless, recent studies on the impacts of these factors on the curative effect of kanglaite adjuvant therapy remain insufficient, and further investigations should be performed.

There are some limitations in our analysis. First, as an important Chinese herbal preparation, kanglaite is mainly used in China, which may result in unavoidable regional bias and subsequently influence the clinical application of kanglaite worldwide. Currently, four clinical trials in the U.S.A. in which malignancies are being treated by kanglaite in conjunction with conventional regimens have been registered on ClinicalTrials.gov (one for prostate cancer, NCT01483586; one for NSCLC, NCT01640730; one for PC, NCT00733850; and one for refractory solid tumors, NCT00031031). Schwartzberg et al. (NCT00733850) [7] reported that compared with gemcitabine alone, kanglaite injection combined with gemcitabine significantly improved the progression-free survival, median OS and QoL of PC patients. Regardless, to date, no trial meeting our inclusion criteria has been published outside China. We will continue to pay close attention to global studies in further analyses. Second, confounding factors such as smoking and alcohol history may have an impact on the efficacy of kanglaite-mediated therapy. However, our data were extracted from publications where this information was not sufficiently provided. Therefore, based on currently available literature, there are insufficient data to perform a statistical analysis to evaluate correlations. We will focus on this concern in future studies. Third, as the sources of our data were published articles instead of raw records from clinical trials, analytical bias may exist. Finally, significant heterogeneity among the included trials was found in some cases, which may be due to the different ages of the HCC patients, tumor stages and durations of treatment. However, based on the currently available literature, there are insufficient data to perform more statistical analyses to evaluate these correlations.

## Conclusions

In conclusion, the findings of this meta-analysis indicate that kanglaite combined with CT is effective in treating advanced HCC. The clinical application of kanglaite not only clearly enhances the therapeutic effects of CT but also effectively improves the QoL and immune function of HCC patients. However, the low quality of some of the included publications increases the risk of bias, which to some extent affects the reliability of the research. The clinical efficacy of kanglaite-mediated adjuvant therapy for advanced HCC still needs to be verified in methodologically rigorous trials.

## Supplementary Material

Supplementary Figures S1-S2 Tables S1-S3

## Abbreviations

AFP: α-fetoprotein
CI: confidence interval
CNKI: China National Knowledge Infrastructure
CT: conventional treatment
DCR: disease control rate
HCC: hepatocellular carcinoma
KPS: Karnofsky performance score
NSCLC: non-small cell lung cancer
OR: odds ratio
ORR: overall response rate
OS: overall survival
PC: pancreatic cancer
QoL: quality of life
SFDA: Chinese State Food and Drug Administration
TACE: transcatheter arterial chemoembolization
TAE: transhepatic arterial embolization
