# Supplementary material for: The clinical efficacy and safety of kanglaite adjuvant therapy in the treatment of advanced hepatocellular carcinoma: A PRISMA-compliant meta-analysis
Source: Biosci Rep. 2019 Nov 26;39(11):BSR20193319. doi: 10.1042/BSR20193319 (PMC6881210; doi:10.1042/BSR20193319)
Supplement: Supplementary Figures S1-S2 Tables S1-S3 [file BSR-2019-3319_supp.pdf]

| Study or Subgroup | Experimental Events | Experimental Total | Control Events | Control Total | Weight | Odds Ratio M-H, Random, 95% CI |
|---|---|---|---|---|---|---|
| **20.1.1 Clinical symptoms** | | | | | | |
| Hu JB 2003 | 27 | 31 | 13 | 25 | 3.7% | 6.23 [1.68, 23.11] |
| Jiang YB 2006 | 85 | 105 | 13 | 51 | 5.7% | 12.42 [5.60, 27.54] |
| Li M 2015 | 14 | 24 | 6 | 23 | 3.9% | 3.97 [1.15, 13.64] |
| Liang SM 2006 | 25 | 31 | 16 | 25 | 4.0% | 2.34 [0.70, 7.85] |
| Wu DH 2009 | 22 | 30 | 10 | 30 | 4.4% | 5.50 [1.81, 16.68] |
| Yang T 2013 | 39 | 60 | 10 | 30 | 5.1% | 3.71 [1.47, 9.38] |
| **Subtotal (95% CI)** | | **281** | | **184** | **26.8%** | **5.36 [3.21, 8.94]** |
| Total events | 212 | | 68 | | | |
| Heterogeneity: $Tau^2 = 0.11$; $Chi^2 = 6.92$, df = 5 (P = 0.23); $I^2 = 28\%$ | | | | | | |
| Test for overall effect: Z = 6.42 (P < 0.00001) | | | | | | |
| **20.1.2 Appetite** | | | | | | |
| Jiang YB 2006 | 67 | 84 | 7 | 43 | 4.9% | 20.27 [7.69, 53.41] |
| Lv DZ 2004 | 16 | 35 | 14 | 34 | 5.0% | 1.20 [0.46, 3.12] |
| Qin GY 1998 | 6 | 14 | 2 | 14 | 2.4% | 4.50 [0.72, 28.15] |
| Ye X 2003 | 16 | 18 | 9 | 17 | 2.5% | 7.11 [1.23, 40.98] |
| Zhu XF 2006 | 25 | 30 | 12 | 28 | 4.0% | 6.67 [1.97, 22.52] |
| **Subtotal (95% CI)** | | **181** | | **136** | **18.8%** | **5.50 [1.72, 17.61]** |
| Total events | 130 | | 44 | | | |
| Heterogeneity: $Tau^2 = 1.30$; $Chi^2 = 16.93$, df = 4 (P = 0.002); $I^2 = 76\%$ | | | | | | |
| Test for overall effect: Z = 2.87 (P = 0.004) | | | | | | |
| **20.1.3 Hepatalgia** | | | | | | |
| Jiang YB 2006 | 47 | 77 | 15 | 42 | 5.8% | 2.82 [1.29, 6.15] |
| Lv DZ 2004 | 22 | 36 | 16 | 37 | 5.1% | 2.06 [0.81, 5.25] |
| Ye X 2003 | 18 | 19 | 13 | 17 | 1.7% | 5.54 [0.55, 55.49] |
| Zhu XF 2006 | 17 | 25 | 6 | 21 | 3.8% | 5.31 [1.50, 18.84] |
| **Subtotal (95% CI)** | | **157** | | **117** | **16.4%** | **2.95 [1.74, 5.00]** |
| Total events | 104 | | 50 | | | |
| Heterogeneity: $Tau^2 = 0.00$; $Chi^2 = 1.69$, df = 3 (P = 0.64); $I^2 = 0\%$ | | | | | | |
| Test for overall effect: Z = 4.03 (P < 0.0001) | | | | | | |
| **20.1.4 Abdominal distention** | | | | | | |
| Jiang YB 2006 | 64 | 86 | 9 | 41 | 5.3% | 10.34 [4.27, 25.04] |
| Lv DZ 2004 | 20 | 38 | 14 | 38 | 5.2% | 1.90 [0.76, 4.76] |
| Qin GY 1998 | 9 | 15 | 4 | 15 | 3.0% | 4.13 [0.88, 19.27] |
| Zhu XF 2006 | 6 | 16 | 5 | 18 | 3.3% | 1.56 [0.37, 6.62] |
| **Subtotal (95% CI)** | | **155** | | **112** | **16.7%** | **3.52 [1.33, 9.31]** |
| Total events | 99 | | 32 | | | |
| Heterogeneity: $Tau^2 = 0.62$; $Chi^2 = 8.58$, df = 3 (P = 0.04); $I^2 = 65\%$ | | | | | | |
| Test for overall effect: Z = 2.54 (P = 0.01) | | | | | | |
| **20.1.5 Fatigue** | | | | | | |
| Jiang YB 2006 | 68 | 93 | 8 | 44 | 5.3% | 12.24 [5.01, 29.89] |
| Lv DZ 2004 | 26 | 37 | 19 | 37 | 5.0% | 2.24 [0.86, 5.82] |
| Qin GY 1998 | 7 | 16 | 3 | 17 | 2.9% | 3.63 [0.74, 17.81] |
| Zhu XF 2006 | 11 | 16 | 6 | 16 | 3.2% | 3.67 [0.85, 15.84] |
| **Subtotal (95% CI)** | | **162** | | **114** | **16.4%** | **4.60 [1.89, 11.22]** |
| Total events | 112 | | 36 | | | |
| Heterogeneity: $Tau^2 = 0.46$; $Chi^2 = 6.91$, df = 3 (P = 0.07); $I^2 = 57\%$ | | | | | | |
| Test for overall effect: Z = 3.35 (P = 0.0008) | | | | | | |
| **20.1.6 Jaundice** | | | | | | |
| Jiang YB 2006 | 11 | 24 | 4 | 9 | 3.0% | 1.06 [0.23, 4.94] |
| Ye X 2003 | 5 | 7 | 4 | 8 | 1.9% | 2.50 [0.29, 21.40] |
| **Subtotal (95% CI)** | | **31** | | **17** | **4.9%** | **1.42 [0.41, 4.95]** |
| Total events | 16 | | 8 | | | |
| Heterogeneity: $Tau^2 = 0.00$; $Chi^2 = 0.41$, df = 1 (P = 0.52); $I^2 = 0\%$ | | | | | | |
| Test for overall effect: Z = 0.55 (P = 0.59) | | | | | | |
| **Total (95% CI)** | | **967** | | **680** | **100.0%** | **4.19 [3.00, 5.86]** |
| Total events | 673 | | 238 | | | |
| Heterogeneity: $Tau^2 = 0.35$; $Chi^2 = 48.87$, df = 24 (P = 0.002); $I^2 = 51\%$ | | | | | | |
| Test for overall effect: Z = 8.40 (P < 0.00001) | | | | | | |
| Test for subgroup differences: $Chi^2 = 5.57$, df = 5 (P = 0.35), $I^2 = 10.3\%$ | | | | | | |

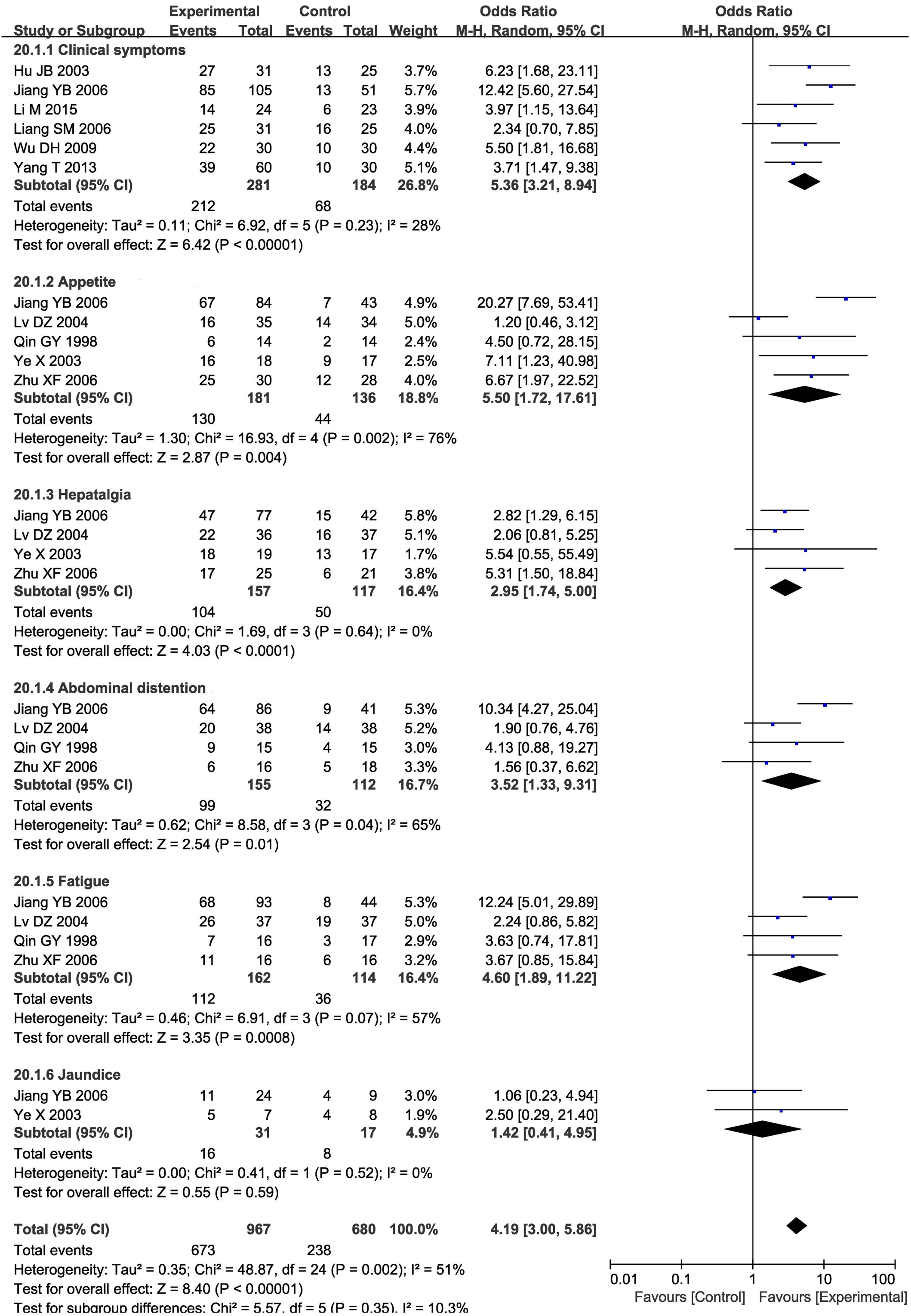

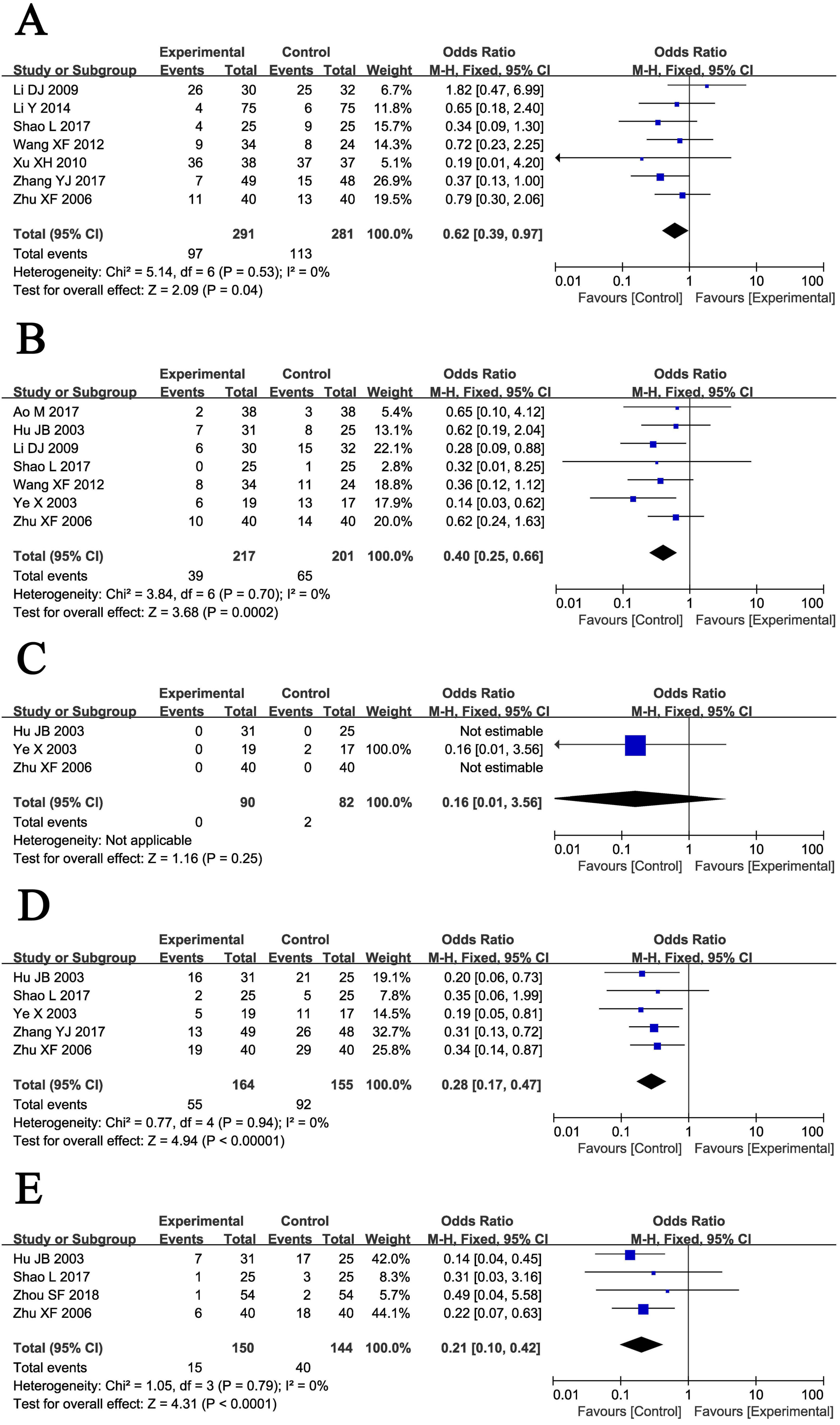
A
Experimental | Control | Odds Ratio | Odds Ratio
Study or Subgroup | Events | Total | Events | Total | Weight | M-H, Fixed, 95% CI | M-H, Fixed, 95% CI
Li DJ 2009 | 26 | 30 | 25 | 32 | 6.7% | 1.82 [0.47, 6.99]
Li Y 2014 | 4 | 75 | 6 | 75 | 11.8% | 0.65 [0.18, 2.40]
Shao L 2017 | 4 | 25 | 9 | 25 | 15.7% | 0.34 [0.09, 1.30]
Wang XF 2012 | 9 | 34 | 8 | 24 | 14.3% | 0.72 [0.23, 2.25]
Xu XH 2010 | 36 | 38 | 37 | 37 | 5.1% | 0.19 [0.01, 4.20]
Zhang YJ 2017 | 7 | 49 | 15 | 48 | 26.9% | 0.37 [0.13, 1.00]
Zhu XF 2006 | 11 | 40 | 13 | 40 | 19.5% | 0.79 [0.30, 2.06]
Total (95% CI) | 291 | 281 | 100.0% | 0.62 [0.39, 0.97]
Total events | 97 | 113
Heterogeneity: Chi² = 5.14, df = 6 (P = 0.53); I² = 0%
Test for overall effect: Z = 2.09 (P = 0.04)
0.01 0.1 1 10 100
Favours [Control] Favours [Experimental]
B
Experimental | Control | Odds Ratio | Odds Ratio
Study or Subgroup | Events | Total | Events | Total | Weight | M-H, Fixed, 95% CI | M-H, Fixed, 95% CI
Ao M 2017 | 2 | 38 | 3 | 38 | 5.4% | 0.65 [0.10, 4.12]
Hu JB 2003 | 7 | 31 | 8 | 25 | 13.1% | 0.62 [0.19, 2.04]
Li DJ 2009 | 6 | 30 | 15 | 32 | 22.1% | 0.28 [0.09, 0.88]
Shao L 2017 | 0 | 25 | 1 | 25 | 2.8% | 0.32 [0.01, 8.25]
Wang XF 2012 | 8 | 34 | 11 | 24 | 18.8% | 0.36 [0.12, 1.12]
Ye X 2003 | 6 | 19 | 13 | 17 | 17.9% | 0.14 [0.03, 0.62]
Zhu XF 2006 | 10 | 40 | 14 | 40 | 20.0% | 0.62 [0.24, 1.63]
Total (95% CI) | 217 | 201 | 100.0% | 0.40 [0.25, 0.66]
Total events | 39 | 65
Heterogeneity: Chi² = 3.84, df = 6 (P = 0.70); I² = 0%
Test for overall effect: Z = 3.68 (P = 0.0002)
0.01 0.1 1 10 100
Favours [Control] Favours [Experimental]
C
Experimental | Control | Odds Ratio | Odds Ratio
Study or Subgroup | Events | Total | Events | Total | Weight | M-H, Fixed, 95% CI | M-H, Fixed, 95% CI
Hu JB 2003 | 0 | 31 | 0 | 25 | Not estimable
Ye X 2003 | 0 | 19 | 2 | 17 | 100.0% | 0.16 [0.01, 3.56]
Zhu XF 2006 | 0 | 40 | 0 | 40 | Not estimable
Total (95% CI) | 90 | 82 | 100.0% | 0.16 [0.01, 3.56]
Total events | 0 | 2
Heterogeneity: Not applicable
Test for overall effect: Z = 1.16 (P = 0.25)
0.01 0.1 1 10 100
Favours [Control] Favours [Experimental]
D
Experimental | Control | Odds Ratio | Odds Ratio
Study or Subgroup | Events | Total | Events | Total | Weight | M-H, Fixed, 95% CI | M-H, Fixed, 95% CI
Hu JB 2003 | 16 | 31 | 21 | 25 | 19.1% | 0.20 [0.06, 0.73]
Shao L 2017 | 2 | 25 | 5 | 25 | 7.8% | 0.35 [0.06, 1.99]
Ye X 2003 | 5 | 19 | 11 | 17 | 14.5% | 0.19 [0.05, 0.81]
Zhang YJ 2017 | 13 | 49 | 26 | 48 | 32.7% | 0.31 [0.13, 0.72]
Zhu XF 2006 | 19 | 40 | 29 | 40 | 25.8% | 0.34 [0.14, 0.87]
Total (95% CI) | 164 | 155 | 100.0% | 0.28 [0.17, 0.47]
Total events | 55 | 92
Heterogeneity: Chi² = 0.77, df = 4 (P = 0.94); I² = 0%
Test for overall effect: Z = 4.94 (P < 0.00001)
0.01 0.1 1 10 100
Favours [Control] Favours [Experimental]
E
Experimental | Control | Odds Ratio | Odds Ratio
Study or Subgroup | Events | Total | Events | Total | Weight | M-H, Fixed, 95% CI | M-H, Fixed, 95% CI
Hu JB 2003 | 7 | 31 | 17 | 25 | 42.0% | 0.14 [0.04, 0.45]
Shao L 2017 | 1 | 25 | 3 | 25 | 8.3% | 0.31 [0.03, 3.16]
Zhou SF 2018 | 1 | 54 | 2 | 54 | 5.7% | 0.49 [0.04, 5.58]
Zhu XF 2006 | 6 | 40 | 18 | 40 | 44.1% | 0.22 [0.07, 0.63]
Total (95% CI) | 150 | 144 | 100.0% | 0.21 [0.10, 0.42]
Total events | 15 | 40
Heterogeneity: Chi² = 1.05, df = 3 (P = 0.79); I² = 0%
Test for overall effect: Z = 4.31 (P < 0.0001)
0.01 0.1 1 10 100
Favours [Control] Favours [Experimental]

# F

| Study or Subgroup | Experimental Events | Experimental Total | Control Events | Control Total | Weight | Odds Ratio M-H, Fixed, 95% CI |
|---|---|---|---|---|---|---|
| Ao M 2017 | 3 | 38 | 4 | 38 | 14.0% | 0.73 [0.15, 3.50] |
| Li M 2015 | 0 | 24 | 2 | 23 | 9.5% | 0.18 [0.01, 3.86] |
| Lu H 2006 | 19 | 24 | 20 | 24 | 15.9% | 0.76 [0.18, 3.26] |
| Yang T 2013 | 4 | 60 | 6 | 30 | 28.4% | 0.29 [0.07, 1.10] |
| Ye X 2003 | 14 | 19 | 17 | 17 | 19.3% | 0.08 [0.00, 1.48] |
| Yu ZH 2016 | 16 | 20 | 17 | 20 | 12.9% | 0.71 [0.14, 3.66] |
| **Total (95% CI)** | | **185** | | **152** | **100.0%** | **0.43 [0.22, 0.84]** |
| Total events | 56 | | 66 | | | |

Heterogeneity: Chi² = 3.37, df = 5 (P = 0.64); I² = 0%
Test for overall effect: Z = 2.46 (P = 0.01)

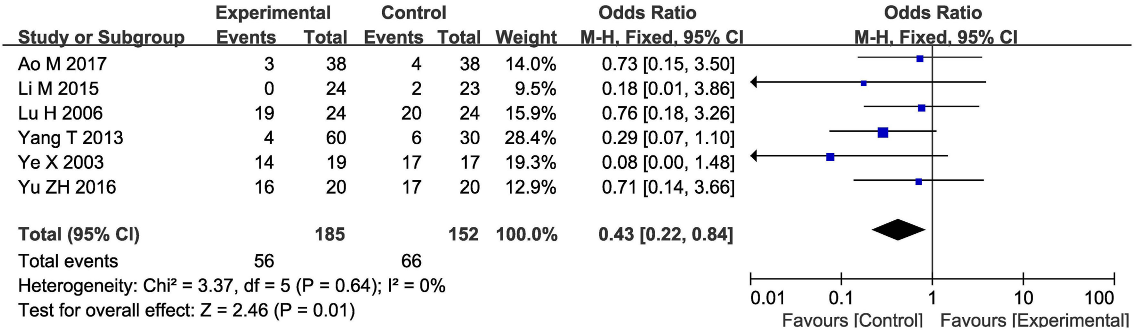


# G

| Study or Subgroup | Experimental Events | Experimental Total | Control Events | Control Total | Weight | Odds Ratio M-H, Fixed, 95% CI |
|---|---|---|---|---|---|---|
| Hu JB 2003 | 6 | 31 | 5 | 25 | 35.1% | 0.96 [0.26, 3.61] |
| Zhu XF 2006 | 7 | 40 | 10 | 40 | 64.9% | 0.64 [0.22, 1.88] |
| **Total (95% CI)** | | **71** | | **65** | **100.0%** | **0.75 [0.33, 1.73]** |
| Total events | 13 | | 15 | | | |

Heterogeneity: Chi² = 0.22, df = 1 (P = 0.64); I² = 0%
Test for overall effect: Z = 0.68 (P = 0.50)

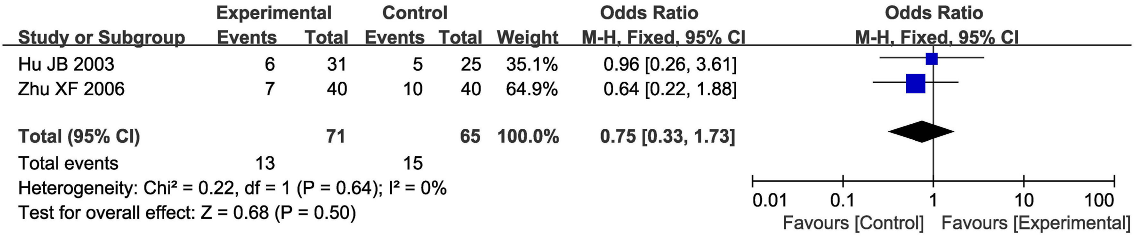


# H

| Study or Subgroup | Experimental Events | Experimental Total | Control Events | Control Total | Weight | Odds Ratio M-H, Fixed, 95% CI |
|---|---|---|---|---|---|---|
| Li DJ 2009 | 19 | 30 | 23 | 32 | 22.7% | 0.68 [0.23, 1.97] |
| Ye X 2003 | 4 | 19 | 10 | 17 | 23.2% | 0.19 [0.04, 0.81] |
| Zhang YJ 2017 | 14 | 49 | 27 | 48 | 54.2% | 0.31 [0.13, 0.72] |
| **Total (95% CI)** | | **98** | | **97** | **100.0%** | **0.37 [0.20, 0.66]** |
| Total events | 37 | | 60 | | | |

Heterogeneity: Chi² = 2.22, df = 2 (P = 0.33); I² = 10%
Test for overall effect: Z = 3.32 (P = 0.0009)

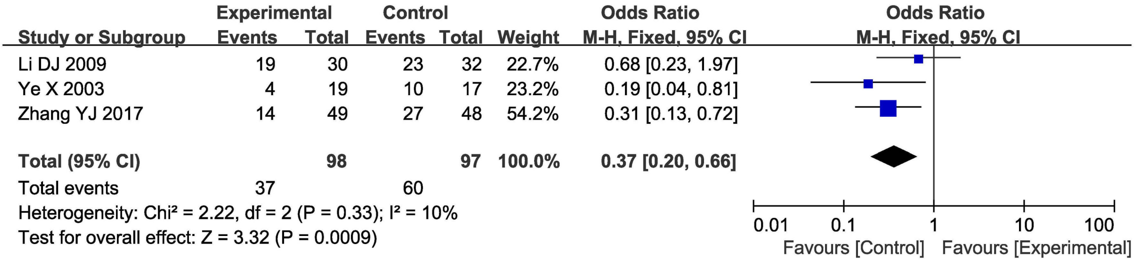


# I

| Study or Subgroup | Experimental Events | Experimental Total | Control Events | Control Total | Weight | Odds Ratio M-H, Fixed, 95% CI |
|---|---|---|---|---|---|---|
| Li Y 2014 | 44 | 75 | 51 | 75 | 84.7% | 0.67 [0.34, 1.30] |
| Yang T 2013 | 3 | 60 | 3 | 30 | 15.3% | 0.47 [0.09, 2.50] |
| **Total (95% CI)** | | **135** | | **105** | **100.0%** | **0.64 [0.34, 1.19]** |
| Total events | 47 | | 54 | | | |

Heterogeneity: Chi² = 0.14, df = 1 (P = 0.71); I² = 0%
Test for overall effect: Z = 1.42 (P = 0.16)

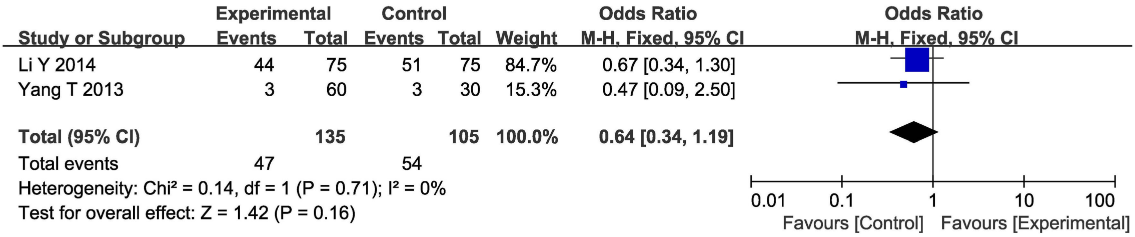


# J

| Study or Subgroup | Experimental Events | Experimental Total | Control Events | Control Total | Weight | Odds Ratio M-H, Fixed, 95% CI |
|---|---|---|---|---|---|---|
| Li Y 2014 | 19 | 75 | 25 | 75 | 82.9% | 0.68 [0.33, 1.38] |
| Zhou SF 2018 | 2 | 54 | 4 | 54 | 17.1% | 0.48 [0.08, 2.74] |
| **Total (95% CI)** | | **129** | | **129** | **100.0%** | **0.64 [0.34, 1.24]** |
| Total events | 21 | | 29 | | | |

Heterogeneity: Chi² = 0.13, df = 1 (P = 0.72); I² = 0%
Test for overall effect: Z = 1.31 (P = 0.19)

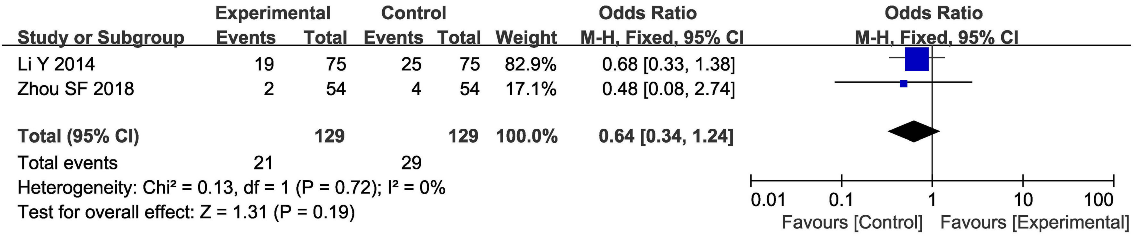

**Supplementary Figure 1.** Forest plot of the comparison of clinical symptoms between the experimental and control groups. Control group, conventional treatments alone group; experimental group, conventional treatments and kanglaite group. A random effects meta-analysis model (Mantel-Haenszel method) was used.

**Supplementary Figure 2.** Forest plot of the comparison of adverse effects, including nausea and vomiting (A), hepatotoxicity (B), nephrotoxicity (C), leucopenia (D), thrombocytopenia (E), gastrointestinal adverse effects (F), anemia (G), fever (H), myelosuppression (I) and alopecia (J), between the experimental and control groups. Control group, conventional treatments alone group; experimental group, conventional treatments and kanglaite group. A fixed effects meta-analysis model (Mantel-Haenszel method) was used.

**Supplement Table 1.** Searching strategy for electronic databases

| Data base | Search Strategy |
|---|---|
| **English database:** PubMed, Cochrane Library, Web of Science, EMBASE and Medline, | **#1.** “liver neoplasm” or “liver carcinoma” “liver cancer” or “liver tumor” or “liver malignant” or “hepatocellular neoplasm” or “hepatocellular carcinoma” or “hepatocellular tumor” or “hepatocellular cancer” or “hepatocellular malignant” or “HC” or “HCC” [Title/Abstract].<br>**#2.** “liver cancer” [MeSH].<br>**#3.** #1 or #2.<br>**#4.** “kanglaite” or “kanglaite capsule” or “kanglaite injection” or “KLT” or “coix seed” or “coix seed capsule” or “coix seed injection” or “yiyiren” [Title/Abstract].<br>**#5. #3** and **#4.**<br>**#6.** limit **#5** to human.<br>**#7.** limit **#6** to (controlled clinical trial).<br>**#8.** limit **#7** to yr=" January 2000-May 2019". |

| | |
|---|---|
| **Chinese database:** China National Knowledge Infrastructure (CNKI), Wanfang Database, Chinese Scientific Journal Database (VIP) and Chinese Biological Medicine Database (CBM) | **#1.** “ganai” or “ganbuzhongliu” “ganxibaoai” or “ganbuzhongwu” or “ganbuzhongkuai” [Title/Keywords].<br>**#2.** “kanglaite” or “kanglaitejiaonang” “kanglaitezhusheye” or “yiyiren” or “yiyirenjiaonang” or “yiyirenzhusheye”[Title/Keywords].<br>**#3. #1** and **#2**<br>**#4.** limit **#3** to human.<br>**#5.** limit **#4** to (controlled clinical trial)<br>**#6.** limit **#5** to yr="2000.1-2019.5" |

**Supplementary Table 2.** The revised and validated version of MINORS

| **Methodological items for non-randomized studies** | Score* |
|---|---|
| **1. A clearly stated aim:** the question addressed should be precise and relevant in the light of available literature. | |
| **2. Inclusion of consecutive patients:** all patients potentially fit for inclusion (satisfying the criteria for inclusion) have been included in the study during the study period (no exclusion or details about the reasons for exclusion). | |
| 3. Prospective collection of data: data were collected according to a protocol established before the beginning of the study. | |
| **4. Endpoints appropriate to the aim of the study:** unambiguous explanation of the criteria used to evaluate the main outcome which should be in accordance with the question addressed by the study. Also, the endpoints should be assessed on an intention-to-treat basis. | |
| **5. Unbiased assessment of the study endpoint:** blind evaluation of objective endpoints and double-blind evaluation of subjective endpoints. Otherwise the reasons for not blinding should be stated. | |
| **6. Follow-up period appropriate to the aim of the study:** the follow-up should be sufficiently long to allow the assessment of the main endpoint and possible adverse events. | |
| **7. Loss to follow up less than 5%:** all patients should be included in the follow up. Otherwise, the proportion lost to follow up should not exceed the proportion experiencing the major endpoint. | |
| **8. Prospective calculation of the study size:** information of the size of detectable difference of interest with a calculation of 95% confidence interval, according to the expected incidence of the outcome event, and information about the level for statistical significance and estimates of power when comparing the outcomes. | |

***Additional criteria in the case of comparative study***

**9. An adequate control group:** having a gold standard diagnostic test or therapeutic intervention recognized as the optimal intervention according to the available published data.

**10. Contemporary groups:** control and studied group should be managed during the same time period (no historical comparison).

**11. Baseline equivalence of groups:** the groups should be similar regarding the criteria other than the studied endpoints. Absence of confounding factors that could bias the interpretation of the results.

**12. Adequate statistical analyses:** whether the statistics were in accordance with the type of study with calculation of confidence intervals or relative risk.

---

*The items are scored 0 (not reported), 1 (reported but inadequate) or 2 (reported and adequate).

**Supplementary Table 3.** Quality assessment of non-randomized comparative studies

| Study | Non-randomized studies | | | | | | | | Additional criteria in comparative study | | | | Total |
|---|---|---|---|---|---|---|---|---|---|---|---|---|---|
| | A | B | C | D | E | F | G | H | I | J | K | L | |
| Li M (2015) | 2 | 1 | 2 | 2 | 2 | 2 | 2 | 0 | 2 | 2 | 2 | 2 | 21 |
| Qin GY (1998) | 2 | 1 | 2 | 2 | 2 | 1 | 2 | 0 | 2 | 2 | 2 | 2 | 20 |
| Qin YT (2001) | 2 | 1 | 2 | 1 | 1 | 1 | 2 | 0 | 2 | 2 | 2 | 2 | 18 |
| Wei QC (2009) | 2 | 1 | 2 | 1 | 1 | 1 | 2 | 0 | 2 | 2 | 2 | 2 | 18 |
| Wu JL (2015) | 2 | 1 | 2 | 2 | 2 | 1 | 2 | 0 | 2 | 2 | 2 | 2 | 20 |
| Xu J (2018) | 2 | 1 | 2 | 2 | 2 | 1 | 2 | 0 | 2 | 2 | 2 | 2 | 20 |
| Yin RR (2009) | 2 | 1 | 2 | 2 | 2 | 1 | 2 | 0 | 2 | 2 | 2 | 2 | 20 |

A: A clearly stated aim; B: Inclusion of consecutive patients; C: Prospective collection of data; D: Endpoints appropriate to the aim of the study; E: Unbiased assessment of the study endpoint; F: Follow-up period appropriate to the aim of the study; G: Loss to follow up less than 5%; H: Prospective calculation of the study size; I: An adequate control group; J: Contemporary groups; K: Baseline equivalence of groups; L: Adequate statistical analyses.

**Notes:** The items are scored 0 (not reported), 1 (reported but inadequate) and 2 (reported and adequate).

# PRISMA 2009 Checklist

| Section/topic | # | Checklist item | Reported on page # |
|---|---|---|---|
| **TITLE** | | | |
| Title | 1 | Identify the report as a systematic review, meta-analysis, or both. | Page 1 (Title) |
| **ABSTRACT** | | | |
| Structured summary | 2 | Provide a structured summary including, as applicable: background; objectives; data sources; study eligibility criteria, participants, and interventions; study appraisal and synthesis methods; results; limitations; conclusions and implications of key findings; systematic review registration number. | Page 2 (Abstract) |
| **INTRODUCTION** | | | |
| Rationale | 3-4 | Describe the rationale for the review in the context of what is already known. | Page 3-4 (Introduction, Fig 1) |
| Objectives | N/A | Provide an explicit statement of questions being addressed with reference to participants, interventions, comparisons, outcomes, and study design (PICOS). | N/A |
| **METHODS** | | | |
| Protocol and registration | N/A | Indicate if a review protocol exists, if and where it can be accessed (e.g., Web address), and, if available, provide registration information including registration number. | N/A |
| Eligibility criteria | 4-5 | Specify study characteristics (e.g., PICOS, length of follow-up) and report characteristics (e.g., years considered, language, publication status) used as criteria for eligibility, giving rationale. | Page 4-5 (Search strategy and selection criteria, Supplementary Table 1) |
| Information sources | 4-5 | Describe all information sources (e.g., databases with dates of coverage, contact with study authors to identify additional studies) in the search and date last searched. | Page 4-5 (Search strategy and selection criteria, Supplementary Table 1) |
| Search | 4-5 | Present full electronic search strategy for at least one database, including any limits used, such that it could be repeated. | Page 4-5 (Search strategy and selection |

| | | | |
|---|---|---|---|
| | | | criteria, Supplementary Table 1) |
| Study selection | 5-6 | State the process for selecting studies (i.e., screening, eligibility, included in systematic review, and, if applicable, included in the meta-analysis). | Page 5-6 (Data extraction and quality assessment, Supplementary Table 2) |
| Data collection process | 5-6 | Describe method of data extraction from reports (e.g., piloted forms, independently, in duplicate) and any processes for obtaining and confirming data from investigators. | Page 5-6 (Data extraction and quality assessment, Supplementary Table 2) |
| Data items | 6 | List and define all variables for which data were sought (e.g., PICOS, funding sources) and any assumptions and simplifications made. | Page 6 (Outcome definition) |
| Risk of bias in individual studies | 5-6 | Describe methods used for assessing risk of bias of individual studies (including specification of whether this was done at the study or outcome level), and how this information is to be used in any data synthesis. | Page 5-6 (Data extraction and quality assessment, Supplementary Table 2) |
| Summary measures | 6 | State the principal summary measures (e.g., risk ratio, difference in means). | Page 6 (Outcome definition) |
| Synthesis of results | 6-7 | Describe the methods of handling data and combining results of studies, if done, including measures of consistency (e.g., $I^2$) for each meta-analysis. | Page 6-7 (Statistical analysis) |



| Section/topic | # | Checklist item | Reported on page # |
|---|---|---|---|

| | | | |
|---|---|---|---|
| Risk of bias across studies | 6-7 | Specify any assessment of risk of bias that may affect the cumulative evidence (e.g., publication bias, selective reporting within studies). | Page 6-7 (Statistical analysis) |
| Additional analyses | 6-7 | Describe methods of additional analyses (e.g., sensitivity or subgroup analyses, meta-regression), if done, indicating which were pre-specified. | Page 6-7 (Statistical analysis) |
| **RESULTS** | | | |
| Study selection | 7 | Give numbers of studies screened, assessed for eligibility, and included in the review, with reasons for exclusions at each stage, ideally with a flow diagram. | Page 7 (Search results, Fig 2) |
| Study characteristics | 7-8 | For each study, present characteristics for which data were extracted (e.g., study size, PICOS, follow-up period) and provide the citations. | Page 7-8 (Patient characteristics, Table 1) |
| Risk of bias within studies | 8 | Present data on risk of bias of each study and, if available, any outcome level assessment (see item 12). | Page 8 (Quality assessment, Fig 3, Supplementary Table 3) |
| Results of individual studies | 7-8 | For all outcomes considered (benefits or harms), present, for each study: (a) simple summary data for each intervention group (b) effect estimates and confidence intervals, ideally with a forest plot. | Page 7-8 (Patient characteristics, Table 1) |
| Synthesis of results | 8-10 | Present results of each meta-analysis done, including confidence intervals and measures of consistency. | Page 8-10 (Therapeutic efficacy assessments, Detection of AFP, QoL assessment, Assessment of the clinical symptoms, Immune function evaluation, Assessment of adverse events Table 2, Fig 4-9, Supplementary |

| | | | |
|---|---|---|---|
| | | | Fig 1 and 2) |
| Risk of bias across studies | 10-11 | Present results of any assessment of risk of bias across studies (see Item 15). | Page 10-11 (Publication bias, Fig 10) |
| Additional analysis | 12 | Give results of additional analyses, if done (e.g., sensitivity or subgroup analyses, meta-regression [see Item 16]). | Page 12 (Sensitivity analysis, Table 3) |
| **DISCUSSION** | | | |
| Summary of evidence | 11-14 | Summarize the main findings including the strength of evidence for each main outcome; consider their relevance to key groups (e.g., healthcare providers, users, and policy makers). | Page 11-14 (Discussion) |
| Limitations | 13-14 | Discuss limitations at study and outcome level (e.g., risk of bias), and at review-level (e.g., incomplete retrieval of identified research, reporting bias). | Page 13-14 (limitations) |
| Conclusions | 14 | Provide a general interpretation of the results in the context of other evidence, and implications for future research. | Page 14 (Conclusion) |
| **FUNDING** | | | |
| Funding | 15 | Describe sources of funding for the systematic review and other support (e.g., supply of data); role of funders for the systematic review. | Page 15 (Funding) |

*From:* Moher D, Liberati A, Tetzlaff J, Altman DG, The PRISMA Group (2009). Preferred Reporting Items for Systematic Reviews and Meta-Analyses: The PRISMA Statement. PLoS Med 6(6): e1000097. doi:10.1371/journal.pmed1000097

For more information, visit: **www.prisma-statement.org**.

# PRISMA 2009 Flow Diagram

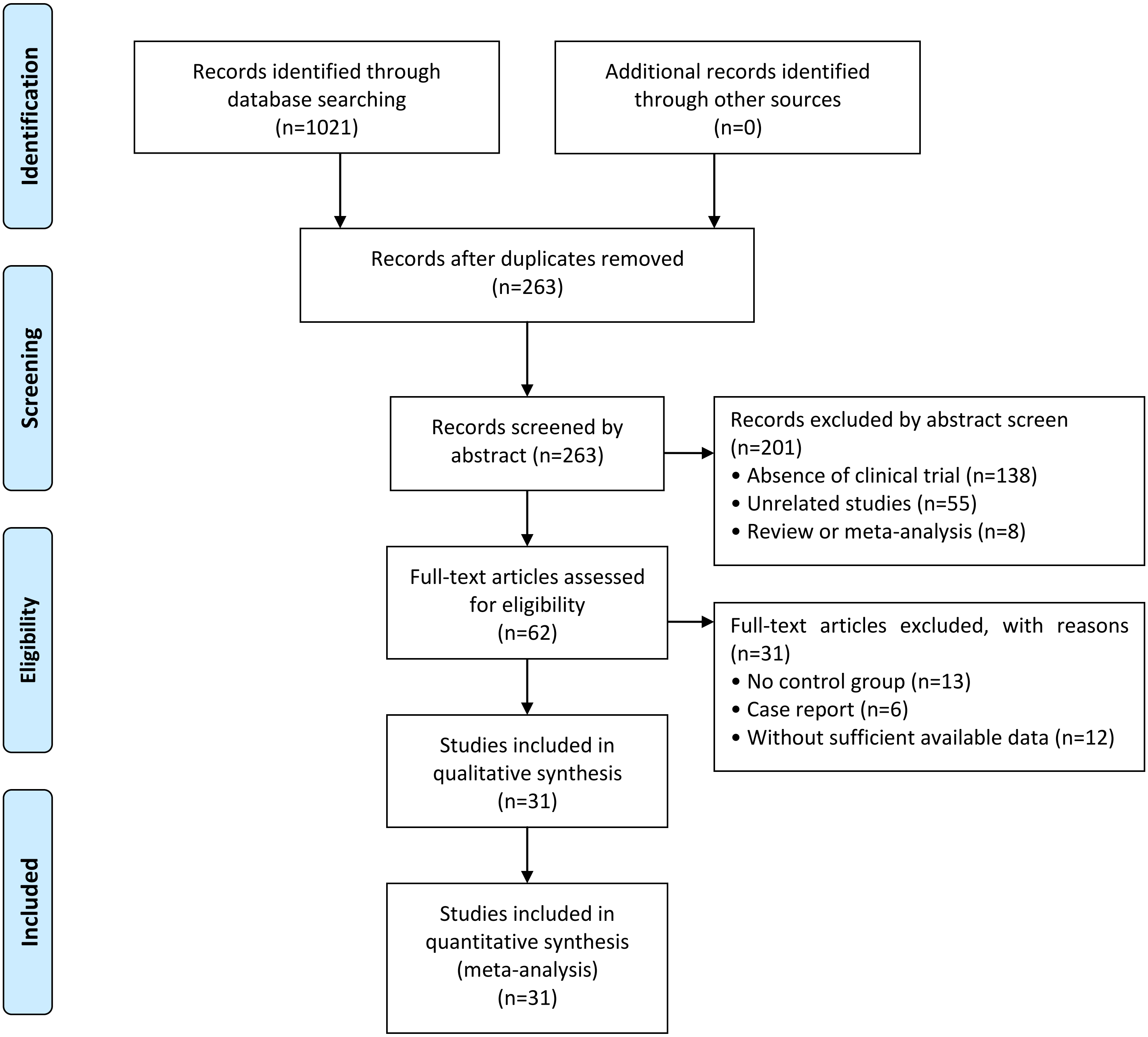


*From:* Moher D, Liberati A, Tetzlaff J, Altman DG, The PRISMA Group (2009). *P*referred *R*eporting *I*tems for *S*ystematic Reviews and *M*eta-*A*nalyses: The PRISMA Statement. PLoS Med 6(7): e1000097. doi:10.1371/journal.pmed1000097

**For more information, visit www.prisma-statement.org.**
